# Distribution of Troy (*Tnfrsf19*) in the Gastric Gland During Postnatal Development: Effects of Early Weaning

**DOI:** 10.1002/cbin.70021

**Published:** 2025-04-09

**Authors:** Isadora Campos Rattes, Kethleen Mesquita da Silva, Patrícia Gama

**Affiliations:** ^1^ Departament of Cell and Developmental Biology, Institute of Biomedical Sciences University of Sao Paulo Sao Paulo Brazil; ^2^ Federal University of Mato Grosso do Sul Campo Grande Brazil

**Keywords:** early‐weaning, stem‐cell, stomach, *Tnfrsf19*, Troy

## Abstract

This study investigates the distribution and role of the stem cell marker Troy (*Tnfrsf19*) in the gastric mucosa of rats during postnatal development and the effects of early weaning. Troy, previously identified as a reserve stem cell marker in adult gastric tissues, is examined across various developmental stages from birth to adulthood. We showed that Troy+ cells are scattered throughout the gastric gland in early postnatal stages, but they become concentrated in the basal portion of the gland as the rats mature. Additionally, early weaning affects Troy expression at its gene and protein levels, altering its distribution in the gastric mucosa. This suggests that early dietary changes may disrupt the organization and function of the secondary stem cell niche in the stomach, potentially impacting gastric gland homeostasis. We also used in silico analysis to compare the molecular functions of Troy+ zymogenic and parietal cells, finding distinct roles in proliferation and secretion. The results underscore the importance of Troy in gastric development and highlight the long‐term impact of early weaning on gastric tissue organization and cell proliferation dynamics.

**Abbreviations**
BMP: bone morphogenetic protein
DAB: 3,3ʹ‐diaminobenzidine tetrahydrochloride
DEPC: diethyl pyrocarbonate
EW: early weaning
ip: intraperitoneal
LGR: leucine‐rich repeat‐containing G‐protein coupled receptor
PMSF: phenylmethylsulfonyl fluoride
pnd: postnatal days
RT: room temperature
S: suckling
TNF: tumor necrosis factor
TROY: tumor necrosis factor receptor superfamily member

## 1 Introduction

In the last decades, several studies tried to identify the location and properties of stem cells in the gastric mucosa of the corpus region, but there is still no evidence of them, neither to confirm a profile nor their existence (Alvina et al. 2023; Han et al. 2019; Karam and Leblond 1993). In those searches, the isthmus‐neck interface was described as the main active proliferative niche for gastric corpus renewal, but, despite this characterization, the complete identification of molecular factors and cellular markers of the niche remains incomplete. Among the markers are Sox2, Mist1, enhancer of the Runx1 gene (eR1), Lrig1, Bmi1, STMN1 and IQGAP3 (Arnold et al. 2011; Choi et al. 2017; Hayakawa et al. 2015; Matsuo et al. 2017, 2021; Han et al. 2019). Although such consensus exists about the isthmus‐neck interface, some studies on stem cells described other markers and a proliferative activity at the base region of the gastric gland, identifying it as a secondary niche (Alvina et al. 2023). One of the markers is the tumor necrosis factor receptor superfamily member 19 (Troy) that was characterized in a reservoir of zymogenic cells. These cells, although differentiated, can undergo cell division, and in vitro, they generate stomach organoids. Under normal conditions, the Troy+ population does not express the Leucine‐rich repeat‐containing G‐protein‐coupled receptor 5 (Lgr5), which is the marker for antral and intestinal stem cells, but it is dependent on the Wnt signaling pathway (Stange et al. 2013). Interestingly, in 2017, the group led by Nick Barker showed that Lgr5 is also expressed in this secondary or reserve niche (zymogenic cells), and they suggested that Lgr5 activation occurs when gastric tissue is injured and it triggers the generation of other gland cell types (Leushacke et al. 2017). So, the main markers of this secondary/reserve proliferative niche at the base would be Troy and Lgr5. As mentioned, Lgr5 is considered the main tag in active stem cells in the small intestine and antral region of the stomach (Barker et al. 2007; Fafilek et al. 2013; Ritsma et al. 2014). As for Troy, its expression is increased in human colorectal cancer cell lines and in mouse intestinal tumors, and it functions as a negative modulator of the Wnt pathway in Lgr5+ intestinal stem cells (Fafilek et al. 2013). The characterization of Troy was carried out in the gastric mucosa of mice, and so far, it is not known how Troy+ cells are distributed and how Troy functions during pre‐ and postnatal stomach development.

In the postnatal period, breastfeeding plays an important role in stomach growth and differentiation (Mesquita da Silva et al. 2021; Osaki et al. 2010; Osaki and Gama 2013; Teles Silva et al. 2020). Previously, we demonstrated that the interruption of breastfeeding through early weaning alters gastric epithelial proliferation (Alvares and Gama 1993; Gama and Alvares 2000; Gama et al. 2000; Ghizoni et al. 2014; Osaki et al. 2011) with an immediate increase of Ki‐67‐proliferative index and mucosal thickness (Mesquita da Silva et al. 2021). Furthermore, the distribution of proliferative cells, that are spread in the mucosa during the suckling period, becomes restricted after early weaning (Mesquita da Silva et al. 2021).

Since both the active and secondary/reserve proliferative gastric niches have been identified in the adult stomach, their development during the postnatal period remains largely unclear. By considering that current knowledge about Troy is restricted to the mature gastric mucosa and injuries, in the current study, we explored the distribution of Troy throughout the ontogeny of the rat gastric mucosa. Additionally, as breastfeeding is part of growth control, we used an early weaning model to analyze the immediate and long‐term effects of dietary changes on this marker.

## 2 Materials and Methods

### 2.1 Animals

Specific‐pathogen‐free Wistar rats were obtained from the Animal Facility at Institute of Biomedical Sciences (University of São Paulo). Pregnant females were kept in individual cages at 22°C under 12:12‐h light/dark cycle with free access to chow (Nuvilab CR‐1, Quimtia). The animals were used in accordance with the certificates approved by the Ethics Committee for the Use of Animals‐ICB (numbers: 18/2015; 115/2017; 4532180222). The delivery day was recorded as zero. Animals of both sexes were used. For early weaning, on the 15th postnatal day (pnd), pups were divided into a control suckling group (S) and an early‐weaning group (EW). EW animals were separated from their mothers and kept in another box containing hydrated powdered chow that was available 24 h a day (Teles Silva et al. 2020). The animals in the control group remained with their mothers until pnd 21. The rats were euthanized at different ages depending on the experimental procedure performed. For analysis of morphology, immunohistochemistry, gene and protein expressions, euthanasia was carried out at 18 and 60 pnd. For detection of Troy during ontogeny, stomach samples were collected at 3, 10, 14, 15, 18, 21, 30, 60, and 120 pnd (*n* = 3 for each age), which characterize different stages of growth and differentiation.

### 2.2 Stomach Collection

In all procedures, at least 3–5 animals/experimental groups were used. The stomach was collected under anesthesia with an ip injection of ketamine hydrochloride and xylazine (Syntec), at an excessive dose (1:1, dose of 0.5 mL/100 g of body mass) between 10 a.m. and 11 a.m. The scraping of the gastric mucosa of corpus region was collected and preserved in RNAlater (Ambion) for gene expression assays or preserved in 0.2 M Tris‐buffered saline (TBS) containing 0.1 M phenylmethanesulfonyl fluoride (PMSF) for protein extraction. In addition, for the morphological study through formalin‐fixed paraffin‐embedding (FFPE), the stomach was collected and fixed in 10% formaldehyde.

### 2.3 Gene Expression—RT‐qPCR

RNA was extracted from the tissue obtained by scraping the gastric mucosa by using the TRIzol method combined with the PureLink RNA purification system (Ambion Kit) according to the manufacturer's specifications. The reactions were conducted in a StepOne Plus thermocycler (AppliedBiosystems), using TaqManProbe assays (AppliedBiosystems). All samples were tested in duplicates. Gene expression was determined using the 2^−^^Δ^ ^Δ^Ct method (Schmittgen and Livak 2008). TaqMan probes were used to detect Troy (*Tnfrsf19‐*Rn01534699_m1) and β‐actin (*Actb‐*Rn00667869_m1).

### 2.4 Immunohistochemistry for Troy—Ontogenic Assay

Histological sections in FFPE from the stomach were obtained from rats at 3, 10, 14, 15, 18, 21, 30, 60, and 120 pnd, and after rehydration in 0.05 M PBS, antigen recovery was performed in 0.01 M Tris‐HCl (pH 9.0) (Merck) (water bath in microwave for 5 min at 600 W power followed by 3 min at 300 W power). After slow cooling (40 min, RT) and washing with 0.05 M PBS, nonspecific binding was blocked with 20% goat serum (30 min) (Jackson ImmunoResearch Laboratories). The sections were incubated overnight at 4°C with rabbit polyclonal antibody against Troy (20 μg/mL, M‐220, Santa Cruz Biotechnology). The reaction was developed with goat anti‐rabbit secondary antibody conjugated to biotin (11 µg/mL, Jackson ImmunoResearch Laboratories,) (2 h, RT), followed by peroxidase‐conjugated streptavidin complex (5 μg/mL, Jackson ImmunoResearch Laboratories) and 0.07% DAB (Sigma‐Aldrich) in 0.02 M TBS containing 3% hydrogen peroxide (Merck) (3 min in the dark box). Slides were counterstained with Mayer's hematoxylin and mounted in Damar's resin. The negative controls were obtained by omission of the primary antibody.

Images were captured using a light microscope (Olympus) (20× magnification), and measurements were taken using the ImagePro‐Plus program (Media Cybernetics). Five fields were considered per animal, and the results were expressed as the number of Troy positive cells per field/animal.

### 2.5 Immunofluorescence for Troy Detection—Effects of Early Weaning

Immunofluorescence reactions for FFPE tissues were performed to analyze the effects of EW on Troy distribution in the gastric gland. The protocol above was followed and the non‐serial sections were incubated with the rabbit polyclonal anti‐Troy (4 μg/mL, overnight, 4°C) (Santa Cruz Biotechnology). The reaction was detected after washing (0.05 M PBS) and incubating with goat anti‐rabbit secondary antibody conjugated to AlexaFluor 488 (4 μg/mL in PBS, 1 h, dark chamber, RT) (Life Technologies). After washing (3×3 min, 0.05 M PBS), the sections were counterstained with DAPI (0.5 μg/mL, Invitrogen) and the slides were mounted in Mowiol (Calbiochem). The negative control of the reaction was obtained by omission of the primary antibody.

The slides were observed under fluorescence microscopy (Axioscope2, Zeiss), using a 40× objective for 18 pnd rats and a 20× objective for 60 pnd animals, both under 10× eyepiece. Images were obtained and were quantified using a digital grid (Zen Blue Software). Five fields were considered per animal, and the results were expressed as the number of Troy positive cells per field/animal. To analyze the distribution in the gland, Troy+ cells were quantified for each region of the mucosa: isthmus, neck, and base.

### 2.6 In Silico Analysis of the Expression Profile of Troy+ Cell Population

Analysis of the expression profile of Troy+ cell population was carried out using a public data set available on the Gene Expression Omnibus (GEO) under the access number GSE44060. The data sets used were derived from parietal‐Troy+ cells and Troy+ zymogenic cells extracted from adult mouse stomachs. The differential expression and geneset (Heatmap) analyses were carried out using the R2: Genomics Analysis and Visualization Platform application, available online at http://r2.amc.nl. The criterion used for differential expression was the Log2 fold change test with a cutoff of 2. Based on the results of the differential analysis, the ontology was created using the Enrich program, considering Gene Ontology 2021.

### 2.7 Western Blot

For protein extraction, the gastric mucosa was homogenized in RIPA buffer. The supernatant was collected for total protein quantification according to the Bradford method (Bradford 1976). The proteins were fractionated in 12% polyacrylamide‐SDS gels (time and volts) and transferred to nitrocellulose membranes (Hybond ECL, GE Healthcare) using transfer buffer (2 h and 4°C, 100 V, 200 mA).

For the immunoblot, membranes were incubated in a blocking solution and incubated (overnight, 4°C) with rabbit polyclonal anti‐Troy primary antibody (5 µg/mL, M220‐Santa Cruz Biotechnology) and with mouse monoclonal anti‐beta actin for loading control. Next, samples were incubated with peroxidase‐conjugated secondary antibodies (GE Healthcare). Development was carried out using the ECL kit (BioRad) and bands were detected under transillumination. The bands were analyzed through densitometry using the public domain software Image J (1.37 v Software, NIH Public Domain).

### 2.8 Statistical Analysis

GraphPad Prism 10.0.3 software (GraphPad Software Inc.) was used for the statistical analysis. The results obtained were grouped according to the experimental groups, represented individually, and the means ± SD was indicated for each group. One‐way ANOVA followed by Tukey tests were used to compare Troy distribution throughout growth and adulthood, and Student's *t*‐test was used to analyze the difference between suckling and EW groups. Distribution of Troy data was analyzed using RStudio software 1.0.153 (RStudio: Integrated Development Environment for R Inc.). *p* < 0.05 was set for all comparisons.

## 3 Results

### 3.1 Identification of Troy in Rat Gastric Mucosa During Postnatal Development

**Figure 1:**
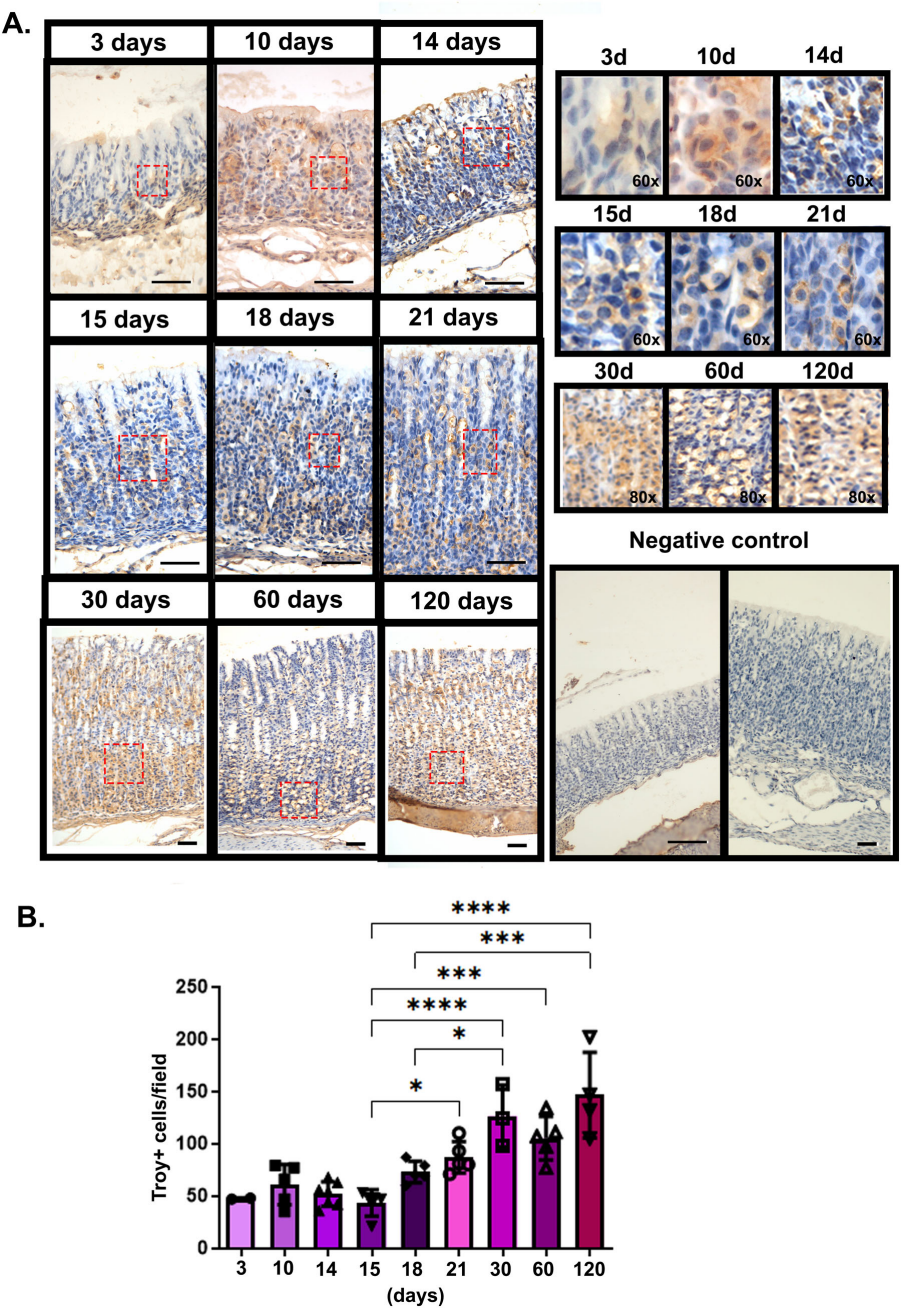
Ontogeny of Troy distribution in the rat gastric mucosa. (A) Left Panel: representative photomicrographs of the gastric mucosa at 3, 10, 14, 15, 18, 21, 30, 60, and 120 pnd. Reactions were developed with DAB and H_2_O_2_ and counterstained with Mayer's hematoxylin. Bar: 50 μm. Right upper panel: digital zoom in for each age and group, providing detailed images of Troy localization. The magnification is indicated by ×*n*. The areas depicted are indicated in the left panel. Right bottom panel: negative controls of Troy reaction by omission of primary antibody. (B) Distribution of the number Troy+ cells/field throughout development and adulthood. Each bar represents the means ± SD of *n* animals, that are individually shown in the associated scatter plot. The results were analyzed by one‐way ANOVA followed by Tukey test. *p < 0.05; ***p < 0.0005; ****p < 0.0001.

Troy is expressed by zymogenic and parietal cells, and it is considered an inactive or reserve stem cell marker (Stange et al. 2013) in adult mice. However, the expression, localization, and function of this marker in the gastric mucosa during postnatal development has not been discussed, and so, currently, we traced Troy expression during gastric gland postnatal growth. It is important to mention that the gastric mucosa of mice and rats are equally organized and can be compared in terms of cell populations, and so, as rats were also being used for early weaning model, we investigated the distribution of Troy in rat samples. By using immunohistochemistry reactions, we detected Troy in the regularly suckling animals at 3, 10, 14, 15, 18, 21, 30, 60, and 120 pnd, and observed its distribution in different regions of the gland. Up to 21 pnd Troy was found to be scattered without a specific region of concentration (Figure 1A). Nonetheless, it is important to consider that up to this age the isthmus, neck and base are not completely defined (Karam and Leblond 1993). and their complete differentiation occurs during weaning period, which in laboratory rodents starts around the 21st pnd. In the adult phase (30 pnd as young adults, 60 and 120 pnd as adults) we identified a higher distribution of Troy in the basal portion of the gland (Figure 1), as it has been reported (Stange et al. 2013). In addition, we found that from the 3rd to the 15th pnd the number of Troy+ cells per field was similar, and from the 18th to the 120th pnd there was an increase of this number (Figure 1B).

### 3.2 Gene Ontology of Troy+ Cells in the Gastric Mucosa

Troy was described in the adult mouse stomach, and after the isolation of Troy+ cells by fluorescence‐activated cell sorting (FACS), the transcriptome analyses indicated that part of these cells were from the zymogenic population and and part of them were parietal cells (Stange et al. 2013). Initial transcriptome analyses focused on gene signatures in zymogenic cells and their roles in restoring the gland after tissue injury. However, the roles of these Troy+ cells during tissue homeostasis have not been fully described. Here, using the microarray data generated by Stange et al. (2013), we carried out an in silico analysis of the gene ontology of Troy+ parietal cells and Troy+ zymogenic cells compared to the gastric gland to identify molecular, cellular, and biological functions. More specifically, we first identified the genes that were highly expressed by Troy+ cells (at least twice increased when compared to all gastric cell populations), and then we compared Troy+ zymogenic cells with Troy+ parietal cells.

#### 3.2.1 Gene Ontology: Molecular Function

**Figure 2:**
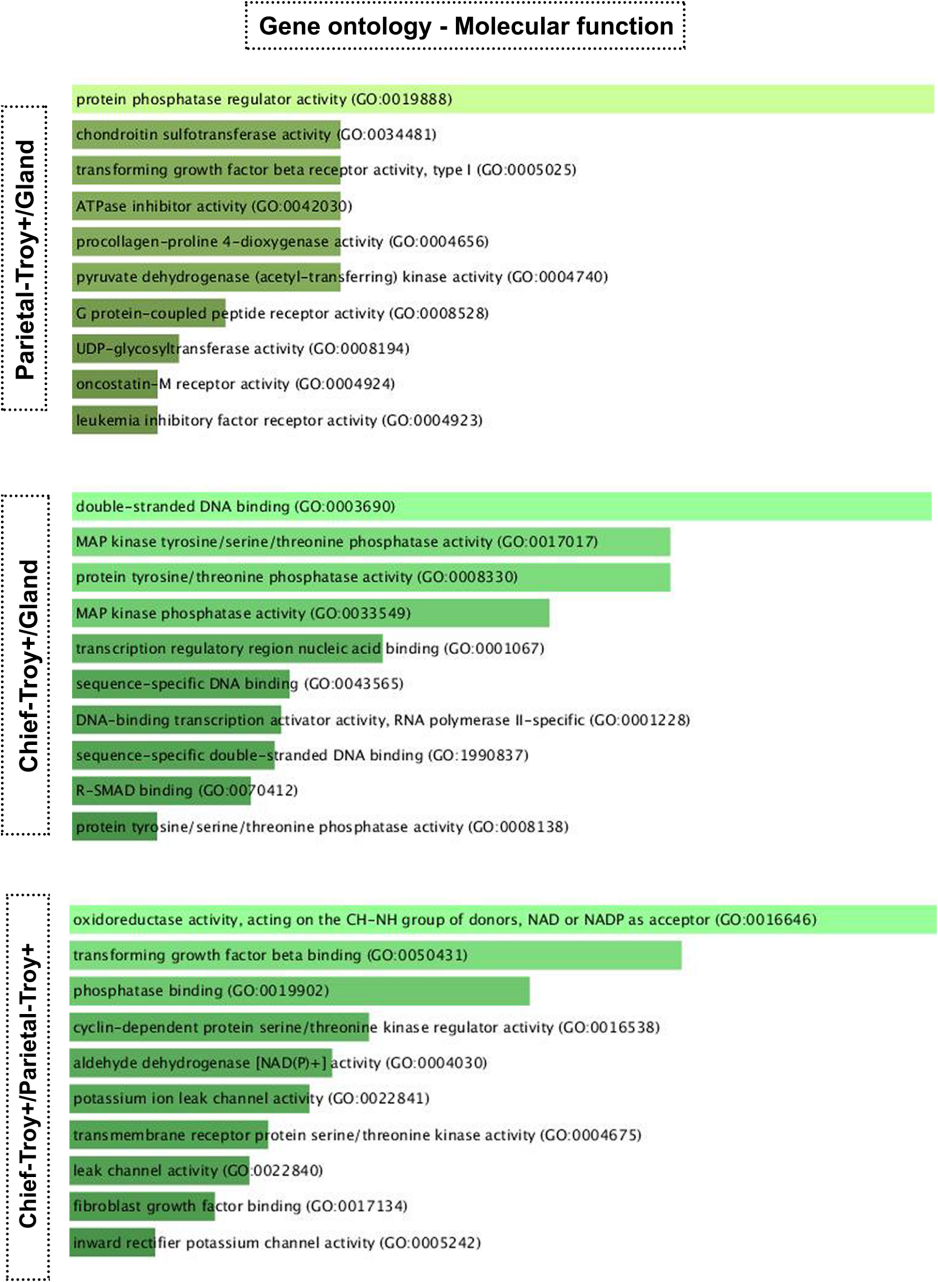
In silico gene ontology for Troy+ cells—molecular functions in parietal and zymogenic cells. The results were analyzed using the R2: Genomics Analysis and Visualization Platform application, and the differential analysis was performed using the Log2 fold change test with a cutoff equal to 2. Based on the results of the differential analysis, the ontology was created using the Enrich program considering Gene Ontology 2021.

Firstly, in relation to the molecular function aspect, which describes activities at the molecular level, we observed that Troy+ parietal cells and Troy+ zymogenic cells are distinct when compared to the gland (Figure 2). For Troy+ parietal cells, the ontology showed genes that are involved in enzymatic regulation of proteins, and for Troy+ zymogenic cells, the genes have roles on cell signaling and DNA regulatory elements. When we compared the two populations (zymogenic Troy+/parietal Troy+), we found that the oxidoreductase activity pathways were highlighted, but the main set of genes indicated the augment of growth factors in zymogenic Troy+ cells.

#### 3.2.2 Gene Ontology: Cellular Component

**Figure 3:**
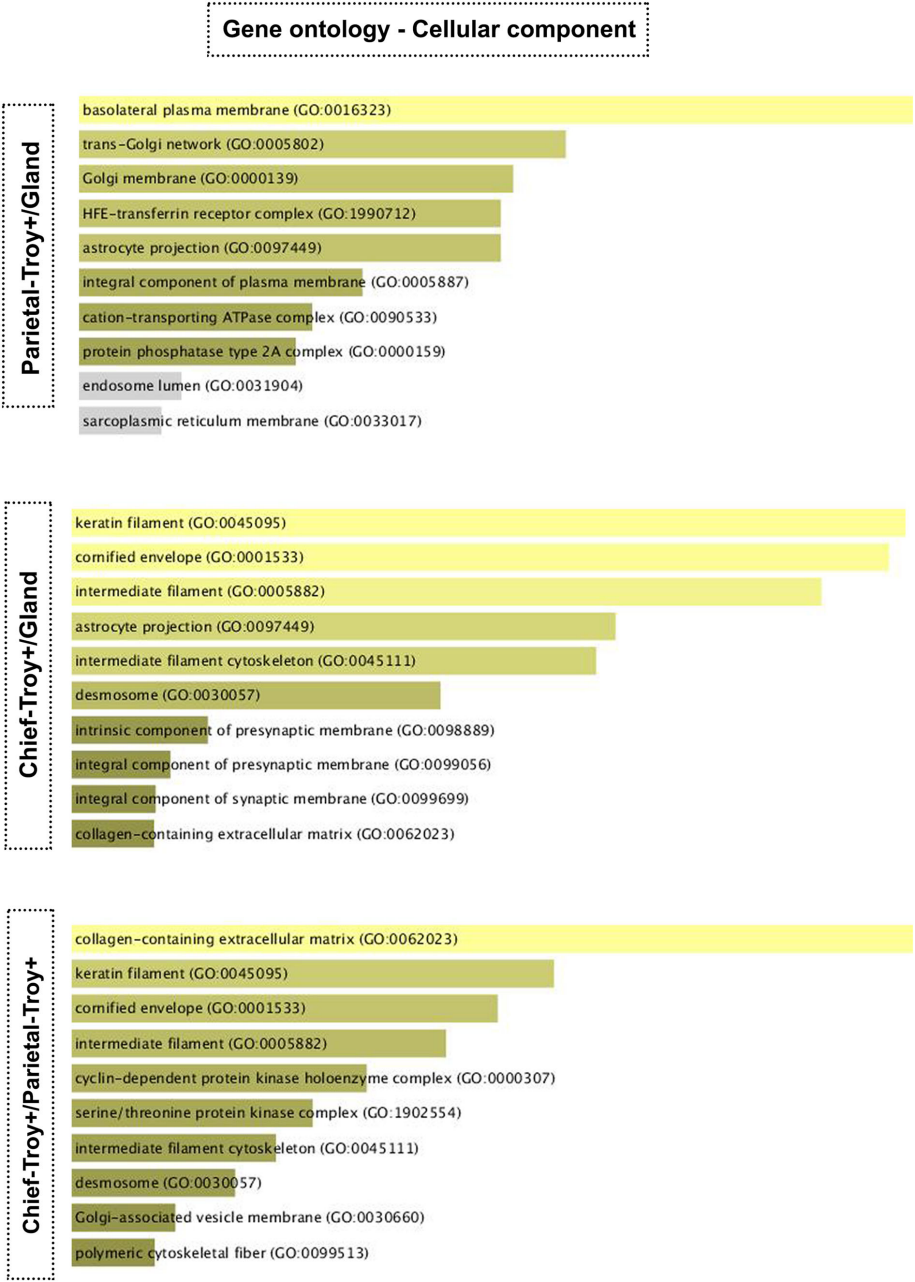
Gene Ontology in the cellular component aspect for parietal cells‐Troy+, zymogenic cells‐Troy+. In silico analysis of the gene ontology of Troy+ parietal cells and Troy zymogenic cells considering the cellular component aspect. The results were analyzed using the R2: Genomics Analysis and Visualization Platform application, and the differential analysis was performed using the Log2 fold change test with a cutoff equal to 2. Based on the results of the differential analysis, the ontology was created using the Enrich program considering Gene Ontology 2021.

Cellular components describe the anatomy of the cell as the location of cellular structures or compartments in which the gene products will perform their functions. After the analysis, we found that the cellular components of Troy+ parietal cells and Troy+ zymogenic cells were distinct when compared to the gland (Figure 3). For Troy+ parietal cells, the ontology highlighted the basolateral plasma membrane and Golgi complex pathways, and for Troy+ zymogenic cells the cellular components were related to the cytoskeleton. When we compared the two populations, the ontology indicated an extracellular matrix element (collagen matrix) that maybe related to the location and arrangement of Troy+ zymogenic cells, and highlighted cytoskeletal elements, which are prevalent in this population (Figure 3).

#### 3.2.3 Gene Ontology: Biological Processes

**Figure 4:**
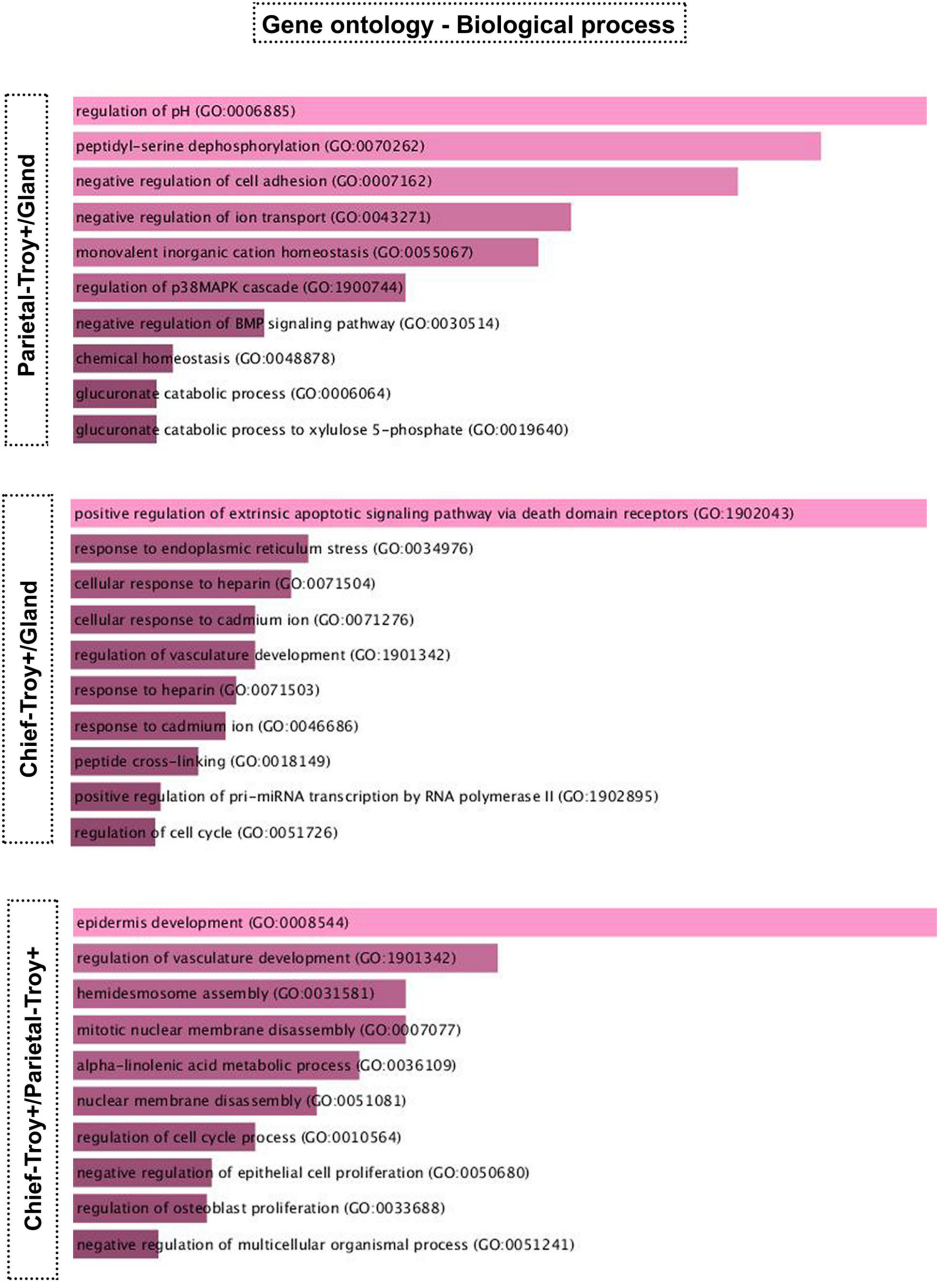
Gene Ontology in the aspect of biological process for parietal cells‐Troy+, zymogenic cells‐Troy+. In silico analysis of the gene ontology of Troy+ parietal cells and Troy zymogenic cells considering the biological process aspect. The results were analyzed using the R2: Genomics Analysis and Visualization Platform application, and the differential analysis was performed using the Log2 fold change test with a cutoff equal to 2. Based on the results of the differential analysis, the ontology was created using the Enrich program considering Gene Ontology 2021.

In the gene ontology focused on the biological processes, the multiple molecular activities were considered, and Troy+ parietal cells and Troy+ zymogenic cells were distinct when compared to the gland, as shown above for the other functions (Figure 4). We observed that Troy+ zymogenic cells are more involved in processes related to the extrinsic apoptotic signaling pathway and cell cycle regulation, whereas Troy+ parietal cells are marked for ion transport processes (pH regulation and control of ion transport). When we compared the two populations, the ontology highlighted the different steps involved in development, especially those connected to cell division and tissue organization.

### 3.3 Effect of Early Weaning on Gene Expression and Troy Protein Level in Gastric Mucosa

**Figure 5:**
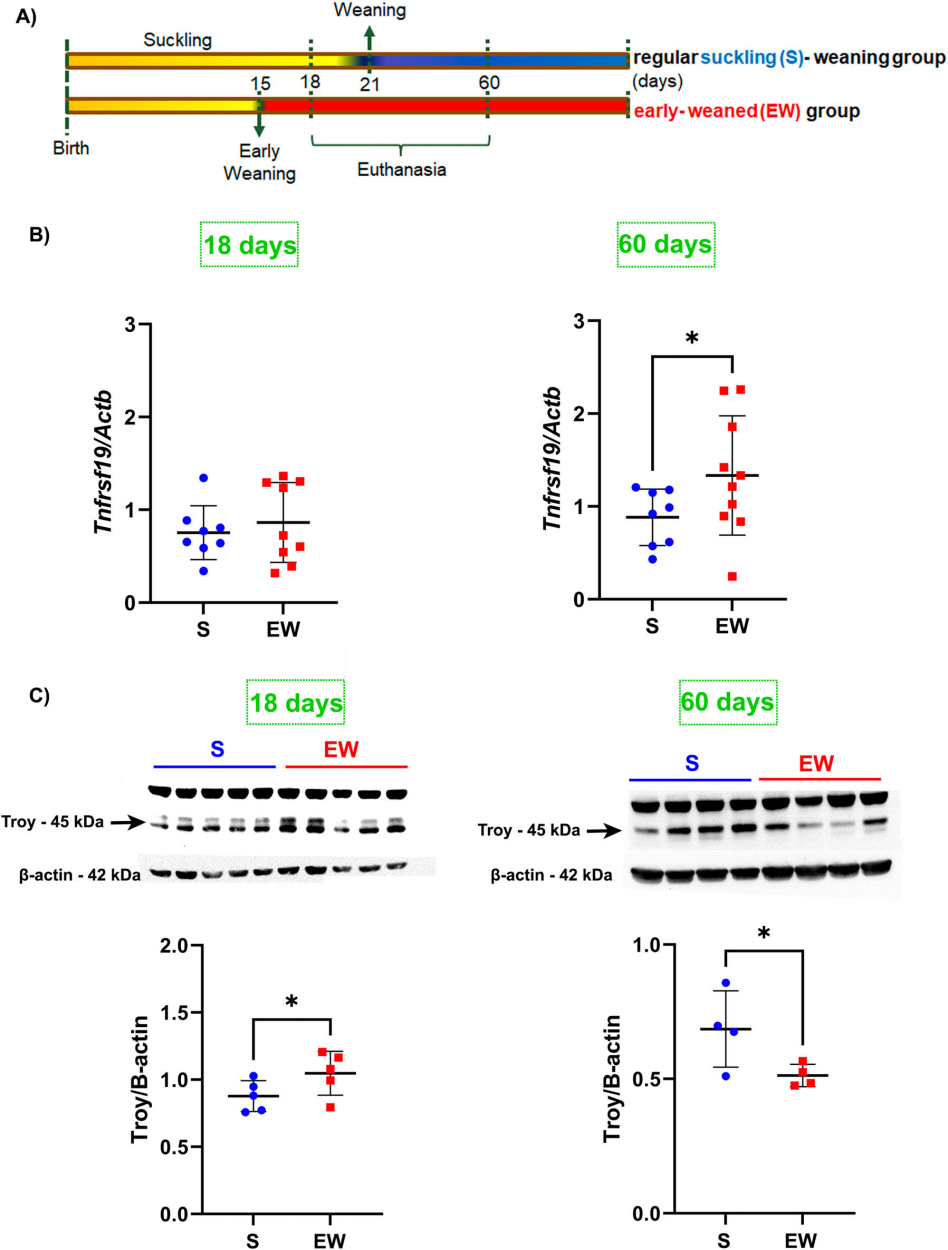
Effects of early weaning at Troy gene expression and protein levels in the stomach. (A) Illustrative diagram for experimental design. (B) Comparison of *Tnfrsf* (TROY) between the suckling (S) and early weaned (EW) groups at 18 and 60 pnd. (C) Representative immunoblots for Troy and the respective endogenous constitutive control used (β‐actin). Indicated are the groups: suckling (S) and subjected to early weaning (EW) at 18 and 60 pnd, samples were distributed on the same 12% SDS‐polyacrylamide gel for each age. The results were obtained from the integrated optical density (IOD) (UA) and compared to the β‐actin reading. The immunoblots were carried out in duplicates, and the bands above Troy are recurrent and appeared in duplicates, possibly due to the use of a polyclonal antibody. Each point represents an animal and the means ± SD are indicated. Results analyzed by Student's *t*‐test with means and ± SD. *p < 0.05.

During postnatal development, breast milk plays an important role in regulating the growth of the gastric mucosa. Previous results showed that in rat pups early milk withdrawal triggers molecular changes in the proliferation and maturation of the gland, which include the expression of important regulatory genes (Teles Silva et al. 2020). We used early weaning model (Figure 5A) to investigate the expression of Troy in the gastric mucosa at 18 and 60 pnd.

We observed that the gene expression of *Tnfrsf19* (Troy‐gene) was not changed by EW at 18 pnd (Figure 5B), but at 60 pnd there was an increase in mRNA, indicating a delayed effect of early weaning on the gastric mucosa. At protein level, EW triggered opposite effects, as we observed an increase and a decrease of Troy in EW rats compared to the control at 18 and 60 pnd, respectively (Figure 5C).

**Figure 6:**
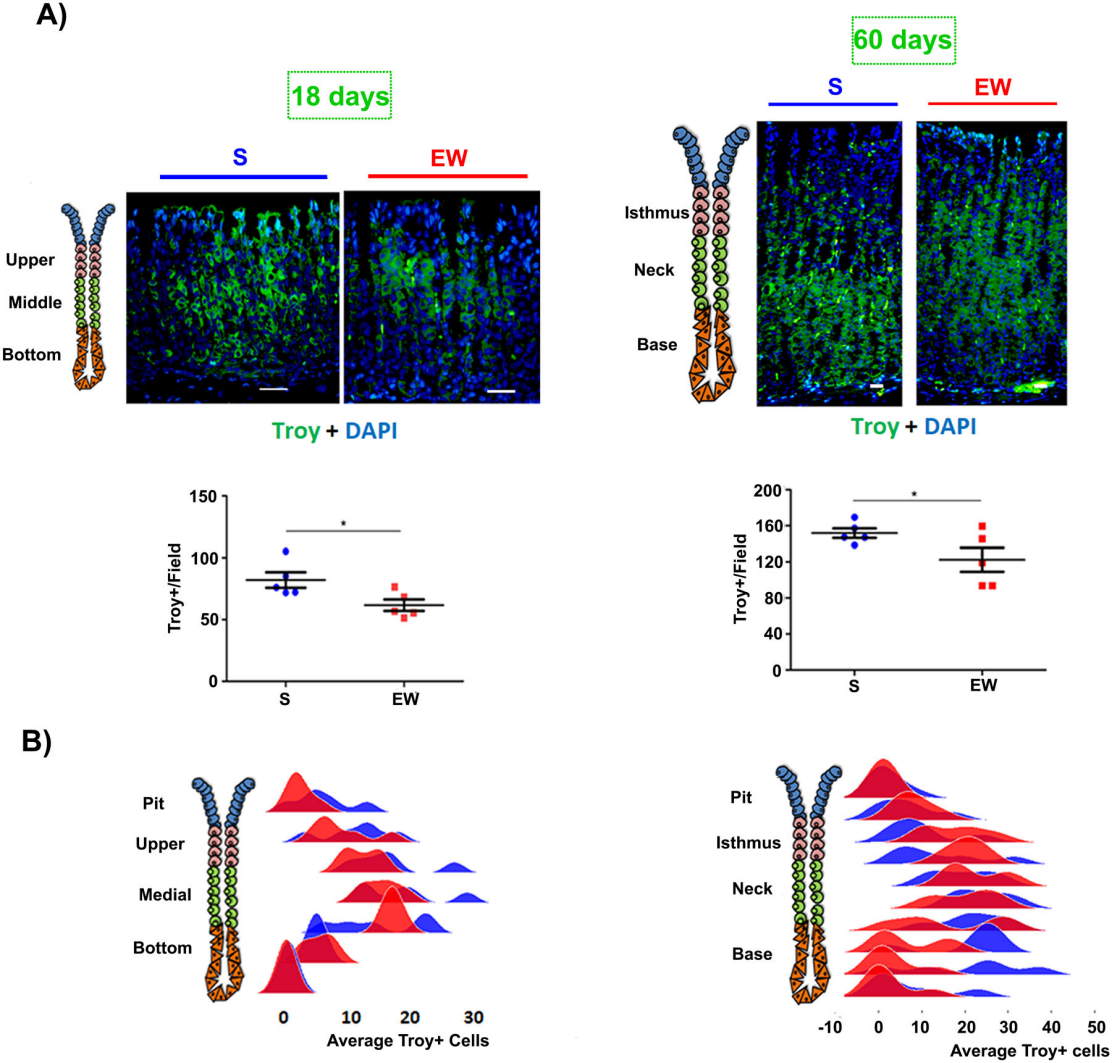
Effect of early weaning on the distribution of Troy+ cells in the gastric mucosa of developing and adult rats. (A) representative images at 18 and 60 pnd. Original magnification: 40×‐18 pnd, 20×‐60 pnd. Nuclei stained with DAPI. Scatter plot with the number of Troy+ cells per field. Results analyzed by Student's *t*‐test with means and ± SD. *N* = 5/group/age. (B) Distribution of Troy+ cells across regions of the gastric gland in 18‐day‐old rats (upper, medial, and bottom) and 60‐day‐old‐animals (isthmus, neck, and base) by Ridgeline Plot graph. Distribution data analyzed using RStudio software 1.0.153. Blue: S‐Group; Red: EW group. *p < 0.05.

In terms of localization in the tissue, at 18 pnd, Troy+ cells were found to be scattered along the gland in the S group (Figure 6A), with a higher concentration in the medial region (Figure 6B, S group in blue), whereas in EW pups (Figure 6A) Troy+ cells distribution was more restricted to the upper and medial regions (Figure 6B, EW group in red). At 60 pnd, Troy+ cells are more abundant at the base of the gland in S rats (Figure 6A), but they can be observed in all other regions as well (Figure 6B), corroborating other studies (Stange et al. 2013). When the effect of EW was investigated in this population, we noticed that the Troy+ cells are more dispersed in the gland (Figure 6A), with a very low concentration at the base (Figure 6B).

To observe whether Troy+ cells were proliferating or differentiated, we compared the Troy distribution with other specific markers (Ki‐67 for proliferation, H+K+ ‐ATPase for parietal cells, and GSII lectin for mucous neck cells). We found that at 18 and 60 pnd, Troy+ cells were not proliferating, and we identified Troy+ cells as parietal cells scattered throughout the gastric gland at both ages (Figure S1). Mucous neck cells did not show double‐labeling with Troy, indicating that in the rat, this population does not express Troy, as previously described in the literature (Stange et al. 2013). As p57 might be involved in the distribution of quiescent zymogenic cells, its localization was also observed in the gastric gland (Figure S2), and labeling was stronger at the base of the gland in the EW group when compared to the S one at 18 pnd, indicating a high protein expression (Figure S2).

## 4 Discussion

The characterization of specific markers for gastric stem cells in the corpus region is still under discussion, despite the various molecules that have been suggested over the last decade (Arnold et al. 2011; Choi et al. 2017; Leushacke et al. 2017; Matsuo et al. 2017; Yoshioka et al. 2019). Han et al. (2019) discussed that for the isthmus region, there is no specific marker, but rather a balance of gene expression that will give rise to the cell types of the gland. For the gland base, two cellular markers were indicated: Lgr5, that was well characterized in the antrum and small intestine (Barker et al. 2007), and Troy (Stange et al. 2013). Although Troy was first described in zymogenic cells, it can also be found in parietal cells (Stange et al. 2013).

Troy (*Tnfrsf19*) is a member of the TNF receptor superfamily and it was originally described in the adult central nervous system, developing hair follicles, and embryonic skin (Hisaoka et al. 2003; Kojima et al. 2000). In the intestine, Troy is expressed in proliferative cells and interacts with Lgr5, inhibiting Wnt signaling in stem cells. Furthermore, its expression is increased in intestinal neoplasms and in human colorectal cancer cell lines (Fafilek et al. 2013). In the stomach, Troy was characterized in zymogenic and parietal cells in adult mice, in conditions when they were capable of entering the cell cycle in response to epithelial injury stimuli. Accordingly, Troy+ cells were described as a secondary niche of stem cells at the base of the gland (Stange et al. 2013).

As previous studies used adult animals, in the current study we evaluated the distribution of Troy in the gastric mucosa in terms of ontogeny, tracking its expression over time. Thus, we examined the presence of Troy in the mucosa from the postnatal phase to adulthood. In immunohistochemistry assays, we detected Troy in regularly suckling animals at 3, 10, 14, 15, 18, 21, 30, 60, and 120 pnd. During the first month, Troy is scattered throughout the gland without a specific concentration region, and in adulthood (30 pnd as young adults; 60 and 120 pnd as adults), we identified a higher concentration of Troy in the basal portion of the gland, as previously reported in the literature (Stange et al. 2013). The average of Troy+ cell distribution remained constant until 15 pnd, and from then on, there was an increase in the population that continued to be detected until adulthood. This process is followed by an increase in the expression of cell differentiation marker genes such as *Atp4b, Bhlha15, Pgc*, and *Gif* (Teles Silva et al. 2020). Therefore, we can suggest that the increase in Troy+ cells in the gastric mucosa is parallel with the cell differentiation occurring during the third week of postnatal life.

Gene ontology analyses showed that Troy+ parietal cells and Troy+ zymogenic cells have distinct functions. Troy+ zymogenic cells exhibited functions more related to proliferation, while Troy+ parietal cells might perform secretion‐related functions with pathways associated with the plasma membrane and Golgi complex. It was not possible to compare Troy‐negative (Troy^Neg^) populations with Troy+ cells, both parietal and zymogenic, as the available data set does not provide information about Troy^Neg^ cell populations, thus, it is not possible to discuss whether a Troy^Neg^ zymogenic cell performs the same functions as a Troy+ one.

In the current study, we investigated gene and protein expression of Troy and we also evaluated its distribution in the gastric gland. As different regulatory levels were observed, we wondered which mechanisms could be involved. Zhu et al. (2022) demonstrated that SNHG8, which is a long noncoding RNA (lncRNA), acts as a molecular scaffold to regulate the expression of Troy. Interestingly, SNHG8 is overexpressed in various tumors and it is associated with cancer cell growth and metastasis of tumors, such as colorectal cancer and gastric carcinoma (Zhu et al. 2022). SNHG8 combines with hnRNPA1 (an RNA‐binding protein from the Heterogeneous nuclear ribonucleoproteins—hnRNPs), participating in processes such as gene expression, RNA maturation, and protein processing. Zhu et al. (2022) demonstrated that SNHG8 regulates Troy, reducing the ability of gastric cancer cells to repair DNA damage (Zhu et al. 2022). Our current results demonstrated that early weaning increased the expression of *Tnfrsf19* (Troy) at 60 pnd when compared to the suckling group, but it decreased the protein content and reduced the number of Troy+ cells at 18 and 60 pnd. Although we did not demonstrate the regulation of Troy by SNHG8‐hnRNPA1, we can suggest that early changes in diet during postnatal development might alter Troy regulation both at the gene expression and protein levels until adulthood through modifications in the SNHG8‐hnRNPA1 axis.

Our previous studies demonstrated that in suckling rats proliferative cells are spread along the gland with a higher concentration in the medial and basal portions (where we find zymogenic cells still in maturation stage), and at 30 pnd, the cells are proliferating in the isthmus region (upper). However, in 18‐day‐old early‐weaned animals, the highest concentration of proliferating cells is in the upper portion of the gland (similar to the adult profile) (Mesquita da Silva et al. 2021). By considering the proliferation results during the first month of postnatal life and the effects of early weaning (Mesquita da Silva et al. 2021), we hypothesized that p57 might also be involved in the proliferative dynamics during postnatal stomach development. CDKN1C/p57 is a cyclin‐dependent kinase inhibitor, involved in different cellular processes, including the control of quiescence of adult hematopoietic and neural stem cells (Furutachi et al. 2013; Matsumoto et al. 2011; Stampone et al. 2018). Lee et al. (2022) showed that p57 also controls the quiescence of adult zymogenic cells. Troy+ zymogenic cells exhibit elevated p57 expression, and Gene Set Enrichment Analysis (GSEA) analyses indicate that these cells are highly enriched for genes typically up regulated in quiescent stem cells. However, after tissue injury, p57 expression decreases and zymogenic cells adopt a proliferative profile capable of mucosal regeneration in mice. Of note, our previous results showed that early weaning induces the precocious differentiation of zymogenic cells at 18 pnd (Teles Silva et al. 2020; Zulian et al. 2017). Currently, we evaluated p57 labeling qualitatively at 18 to 30 pnd in both suckling and early weaning rats. According to our observation, at 18 pnd, the immunostaining was stronger at the base of the gland in the EW group when compared to the S one, suggesting a high protein levels. Besides that, regarding the localization of Troy+ cells, we found that in suckling pups, they are scattered throughout the gland, whereas in EW rats they are more restricted in the isthmus and neck regions. In adult animals, we observed that in the suckling group, most Troy+ cells are at the base, but there are cells in other compartments, while in the EW group, the distribution pattern is diffuse throughout the gland without concentration in any specific region. As we demonstrated, Troy+ were not proliferating and it was expressed by parietal cells. Therefore, by considering the information that p57 controls the quiescence of zymogenic cells, we can hypothesize that the gradual increase in p57 in zymogenic cells during the first month of postnatal life would drive these cells into quiescence in adulthood. Additionally, a dietary change such as early weaning could accelerate this process of p57 increase, leading to zymogenic cell differentiation, quiescence, and distribution in the gland.

## 5 Conclusions

In summary, we demonstrated that the Troy marker of quiescent proliferative cells is expressed in the rat gastric mucosa during postnatal development, acquiring a distribution profile at the base of the gland only after cellular differentiation and stomach maturation. We also described that Troy+ parietal and zymogenic cells have distinct roles in gland homeostasis, and we further showed that early weaning directly affects Troy, altering gene expression, protein level, and distribution along the gland. Such effects occur immediately, and are lately maintained, as were detected the changes up to adulthood, indicating that the early withdrawal of breast milk may destabilize the gland organization of proliferative niches. So we concluded that Troy is important for gastric development and maintenance of the basal proliferative niche, and our results suggest that early weaning has a long‐term impact on gastric tissue organization and cell proliferation dynamics.

## Author Contributions

Isadora Campos Rattes designed the study, planned and ran the experiments, obtained and analyzed the results, and drafted and reviewed the manuscript. Kethleen Mesquita da Silva ran and analyzed the experiments on gene expression and reviewed the manuscript. Patrícia Gama designed the study, obtained the funding, planned the experiments, discussed the results, drafted, and reviewed the manuscript.

## 1 Conflicts of Interest

The authors declare no conflicts of interest.

## Supporting information

Supporting **Figure 1: Detection of Troy in epithelial cells of the gastric gland in suckling and early‐weaned rats.** (A) Detection of double‐positive cells for Troy (AlexaFluor 563–Red) and Ki‐67 (AlexaFluor 488–Green) in the gastric mucosa of animals aged 18 and 60 pnd subjected to early weaning. No overlapping labeling was observed. Representative images of the groups at both ages after digital overlay of serial sections labeled with Troy and Ki‐67. In the digital zoom in, note the non‐overlapping Troy and Ki‐67 labeling. Scale bar: 50 µm. Nuclei stained with DAPI (Blue). (B) Detection of Troy‐positive cells (AlexaFluor 563) and parietal cells (labeled with anti H+/K+‐ATPase–Alexa 488). Double‐labeled cells were identified. In the digital zoom in, note the overlapping Troy and parietal cell labeling. Scale bar: 50 µm. Nuclei stained with DAPI (Blue). (C) Detection of Troy‐positive cells (AlexaFluor 563) and mucous neck cells (labeled with GSII lectin–FITC). No double‐labeled cells were observed at the ages studied. In the digital zoom in, overlapping Troy and GSII lectin was not detected. Scale bar: 50 µm. Nuclei stained with DAPI (Blue). Supporting **Figure 2**: **Distribution of p57‐positive cells in the gastric mucosa.** Immunohistochemistry performed at different ages (18, 21, 25, and 30 days) for p57 detection. Reactions were developed with DAB and H_2_O_2_ and counterstained with Mayer's hematoxylin. Scale bar: 50 µm.

## Data Availability

The data that support the findings of this study are available from the corresponding author upon reasonable request.
