# Supplementary figures and images for: Distribution of Troy (Tnfrsf19) in the Gastric Gland During Postnatal Development: Effects of Early Weaning
Source: Cell Biol Int. 2025 Apr 9;49(7):772–84. doi: 10.1002/cbin.70021 (PMC12172142; doi:10.1002/cbin.70021)

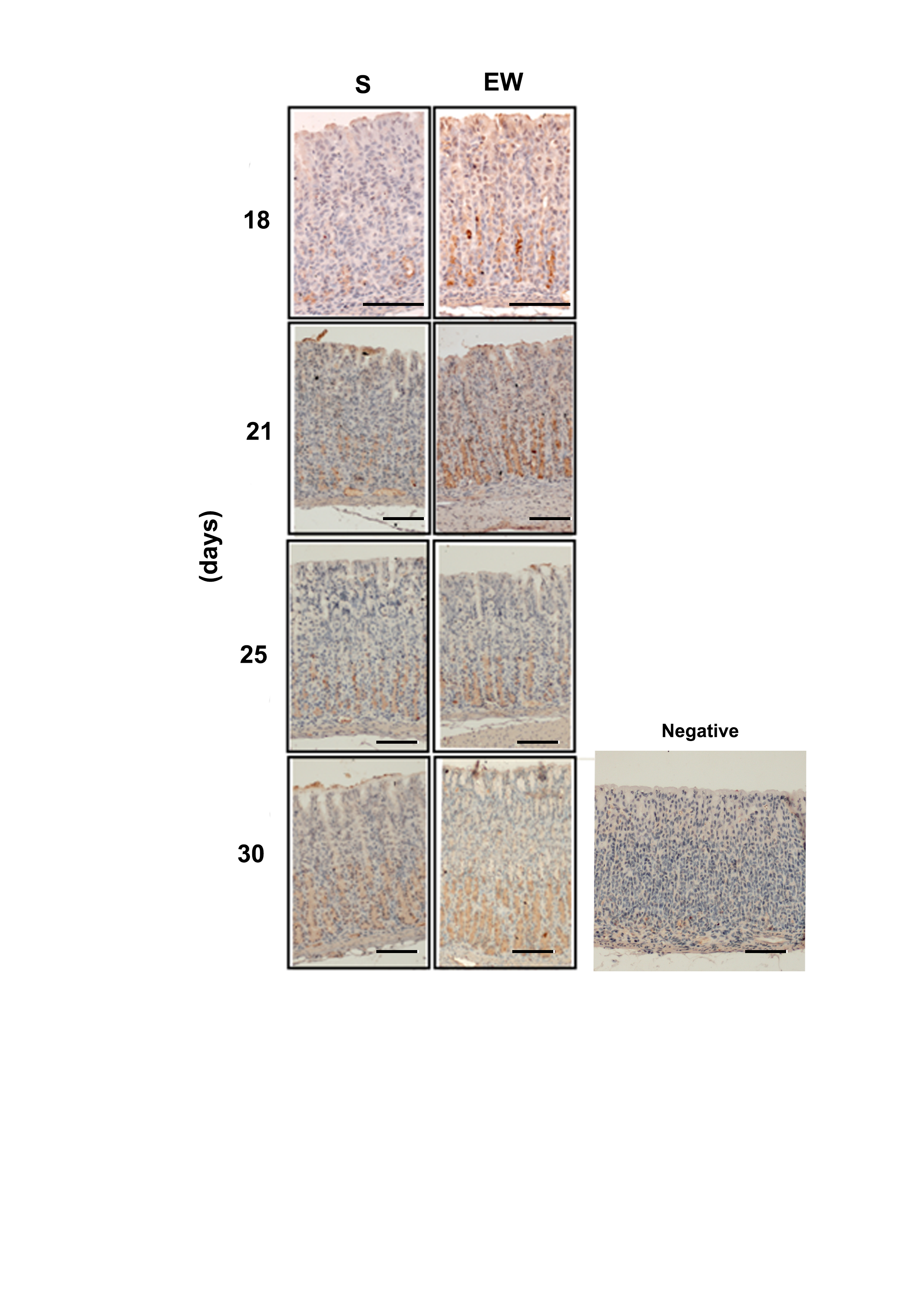

Supplement: Supplementary file 1 — Supporting Figure 1: Detection of Troy in epithelial cells of the gastric gland in suckling and early‐weaned rats. (A) Detection of double‐positive cells for Troy (AlexaFluor 563–Red) and Ki‐67 (AlexaFluor 488–Green) in the gastric mucosa of animals aged 18 and 60 pnd subjected to early weaning. No overlapping labeling was observed. Representative images of the groups at both ages after digital overlay of serial sections labeled with Troy and Ki‐67. In the digital zoom in, note the non‐overlapping Troy and Ki‐67 labeling. Scale bar: 50 µm. Nuclei stained with DAPI (Blue). (B) Detection of Troy‐positive cells (AlexaFluor 563) and parietal cells (labeled with anti H+/K+‐ATPase–Alexa 488). Double‐labeled cells were identified. In the digital zoom in, note the overlapping Troy and parietal cell labeling. Scale bar: 50 µm. Nuclei stained with DAPI (Blue). (C) Detection of Troy‐positive cells (AlexaFluor 563) and mucous neck cells (labeled with GSII lectin–FITC). No double‐labeled cells were observed at the ages studied. In the digital zoom in, overlapping Troy and GSII lectin was not detected. Scale bar: 50 µm. Nuclei stained with DAPI (Blue). Supporting Figure 2: Distribution of p57‐positive cells in the gastric mucosa. Immunohistochemistry performed at different ages (18, 21, 25, and 30 days) for p57 detection. Reactions were developed with DAB and H2O2 and counterstained with Mayer's hematoxylin. Scale bar: 50 µm. [file CBIN-49-772-s001.zip › Rattes_Supplementary Figure 2.tif]
